# Eating Disorder or Disordered Eating; an Interesting Case Study

**DOI:** 10.1192/bjo.2024.677

**Published:** 2024-08-01

**Authors:** Gabriel Michael, Natasha Patel, Charles Stanley

**Affiliations:** Leeds Community Healthcare Trust, Leeds, United Kingdom

## Abstract

**Aims:**

Eating disorders often present as a significant challenge in adolescents; especially with regards to early diagnosis and intervention. This case report explores the complex presentation of a 15-year-old female initially suspected of having an eating disorder. The complexity in this case lies in the differentiation between a formal eating disorder and disordered eating, emphasising the importance of thorough assessment and understanding of the underlying psychological factors.

**Methods:**

This patient presented to the CAMHS eating disorders team having lost 14 kg in 6 weeks. Such an alarming weight loss had triggered the urgent referral and review. These symptoms initially suggested a classic eating disorder. However as the patient spent more time on the physical health ward a comprehensive evaluation revealed underlying issues related to body image, self-esteem, and emotional wellbeing as well as complex family dynamics leading to a diagnosis of disordered eating rather than a specific eating disorder. The multidimensional approach involved collaboration between mental health professionals, paediatricians and dieticians, to address the multifaceted nature of the condition whilst the patient was admitted to a physical health ward.

**Results:**

The case highlights the intricate interplay between physical and psychological factors contributing to disordered eating behaviours in adolescence. Factors such as societal pressures, peer influences, personal expectations as well as dynamics within a family home may all contribute to a distorted relationship with food and body image. Recognising these complexities is crucial for tailored interventions that address the root causes rather than merely focusing on symptomatic relief. We also established in this case the difference in efficacy between utilising aripiprazole vs olanzapine in terms of treatment of anorexic cognitions.

**Conclusion:**

This case study underscores the necessity of a thorough and holistic approach in assessment, diagnosis and management of eating-related concerns in adolescents. By differentiating between eating disorders and disordered eating, healthcare professionals may better accommodate interventions to address the specific needs of the individual. Early identification and comprehensive care, involving medical, nutritional, and psychological components, are essential for promoting healthy relationships with food and preventing the escalation of disordered eating into more severe conditions.

